# Radiation Detector Front-End Readout Chip with Nonbinary Successive Approximation Register Analog-to-Digital Converter for Wearable Healthcare Monitoring Applications

**DOI:** 10.3390/mi15010143

**Published:** 2024-01-17

**Authors:** Hsuan-Lun Kuo, Shih-Lun Chen

**Affiliations:** 1 Department of Engineering and System Science, National Tsing Hua University, Hsinchu 300044, Taiwan; 2 Department of Electronic Engineering, Chung Yuan Christian University, Taoyuan City 320317, Taiwan

**Keywords:** bioelectronics, wearable sensors, healthcare monitoring, radiation detector, nonbinary SAR ADC

## Abstract

A 16-channel front-end readout chip for a radiation detector is designed for portable or wearable healthcare monitoring applications. The proposed chip reads the signal of the radiation detector and converts it into digital serial-out data by using a nonbinary successive approximation register (SAR) analog-to-digital converter (ADC) that has a 1-MS/s sampling rate and 10-b resolution. The minimum-to-maximum differential and integral nonlinearity are measured as −0.32 to 0.33 and −0.43 to 0.37 least significant bits, respectively. The signal-to-noise-and-distortion ratio and effective number of bits are 57.41 dB and 9.24 bits, respectively, for an input frequency of 500 kHz and a sampling rate of 1 MS/s. The SAR ADC has a 38.9-fJ/conversion step figure of merit at the sampling rate of 1 MS/s. The proposed chip can read input signals with peak currents ranging from 20 to 750 μA and convert the analog signal into a 10-bit serial-output digital signal. The input dynamic range is 2–75 pC. The resolution of the peak current is 208.3 nA. The chip, which has an area of 1.444 mm × 10.568 mm, is implemented using CMOS 0.18-μm 1P6M technology, and the power consumption of each channel is 19 mW. This design is suitable for wearable devices, especially biomedical devices.

## 1. Introduction

As integrated chip technology has developed, many large and costly machines have become smaller and less expensive. In the biomedical field, progressive wireless technology and modalities such as electroencephalography [1], electrocardiography [2,3,4,5,6], and magnetic resonance imaging [7] are being integrated into wireless body sensor networks [5,6,8]. Wearable devices are becoming even smaller because digital signal processors and data storage can be outsourced to external devices. Technological developments are leading to considerable benefits in terms of wearable healthcare monitoring.

The use of radiation detector devices in medicine [9,10] is limited by the large size of these devices. Integrated chip technology offers a means of scaling down instruments, and smaller radiation detector devices would have a greater scope of application. Communication within the Internet of Things (IoT) is convenient and rapid, and integrated chip technology enables the use of radiation detector devices in wearable healthcare monitoring and the IoT [11,12,13,14]. The conversion blocks that have been employed to convert analog signals to digital signals are analog-to-digital converters (ADCs) [11,12,13], digital delay-locked loop circuits [14], and time-to-digital converters [15].

In designing wearable healthcare monitoring applications, the objectives are a sampling rate of several megasamples per second and low power consumption.

Pipeline ADCs have high speed, with high sampling rates (~100 MS/s), medium resolution (8–12 b), and high power consumption (dozens of milliwatts). Delta-sigma ADCs have low speed, with sampling rates in the dozens of kilosamples per second, high resolution (12–20 b), and moderate power consumption (several milliwatts). SAR ADCs have medium speed, with sampling rates of several megasamples per second, moderate resolution (8–12 b), and low power consumption (dozens of microwatts). SAR ADCs are thus more suitable than pipelined ADCs or delta-sigma ADCs for wearable healthcare monitoring applications.

**Figure 1:**
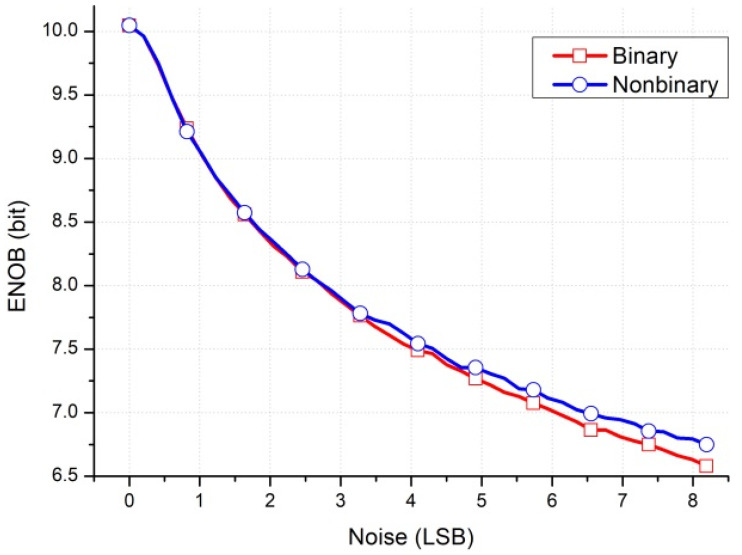
ENOB of binary and nonbinary SAR ADCs with a noise effect in Matlab R2017b simulations.

**Figure 2:**
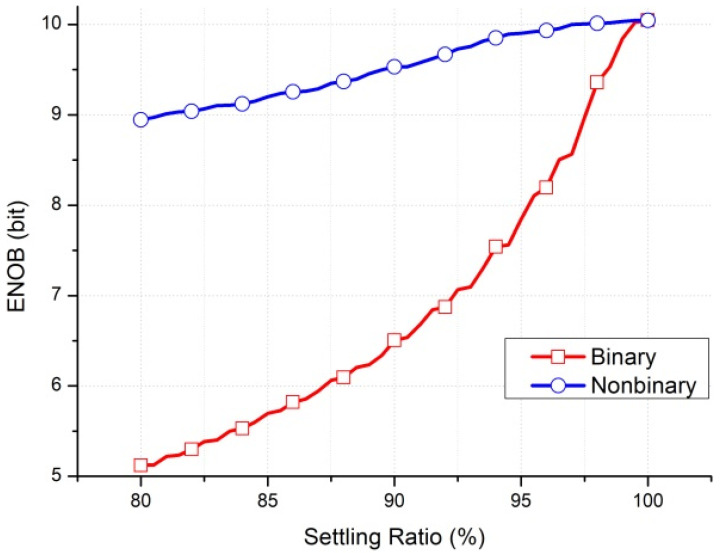
ENOB of binary and nonbinary SAR ADCs versus DAC settling ratio in Matlab simulations.

This study proposes a front-end readout circuit for a radiation detector; this circuit contains a nonbinary successive approximation register (SAR) ADC [16]. In radiation applications, a particle can cause bit flips in digital circuits and voltage spikes in analog circuits. These flips and spikes can be considered a noise effect. Because of its nonbinary structure, the SAR ADC has a correction mechanism that can minimize the effect of the digital-to-analog converter (DAC), settling incomplete errors and the effects of noise from the DAC and comparator [17]. The results of simulations are depicted in Figure 1 and Figure 2. Figure 1 presents a comparison of the effective number of bits (ENOB) for a binary SAR ADC versus a nonbinary SAR ADC when some noise is present in the comparator input. The ENOB of the nonbinary SAR ADC is 0.17 b higher than that of the binary SAR ADC with an 8-least-significant-bit (LSB) noise effect. Figure 2 presents a comparison of the ENOB for binary and nonbinary SAR ADCs for various settling ratios. The settling ratio means that the ratio of DAC voltage settles to the correct value. The ENOB of the nonbinary SAR is still 9 b when the DAC settling ratio is 80%. The ENOB of the binary SAR ADC is 9 b at a settling ratio of 97.5%, whereas at an 80% settling ratio, it is only 5.12 b.

**Figure 3:**
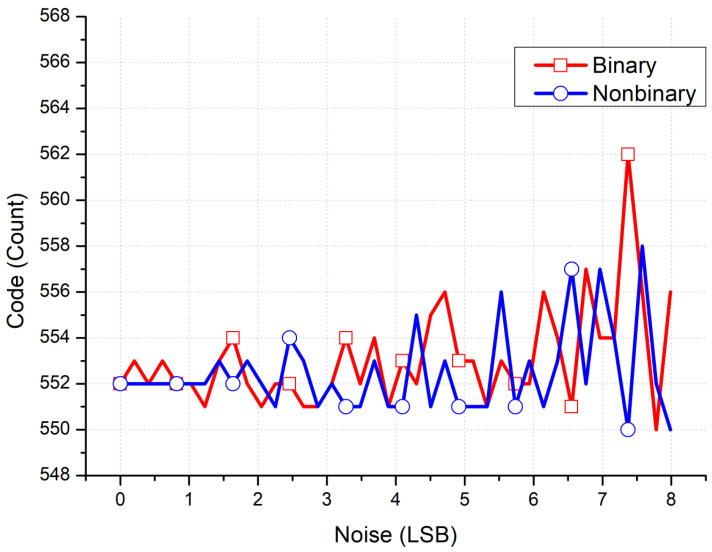
Conversion error versus noise for a radiation detector front-end readout chip containing a binary versus nonbinary SAR ADC with DAC incomplete settling in Matlab simulations.

**Figure 4:**
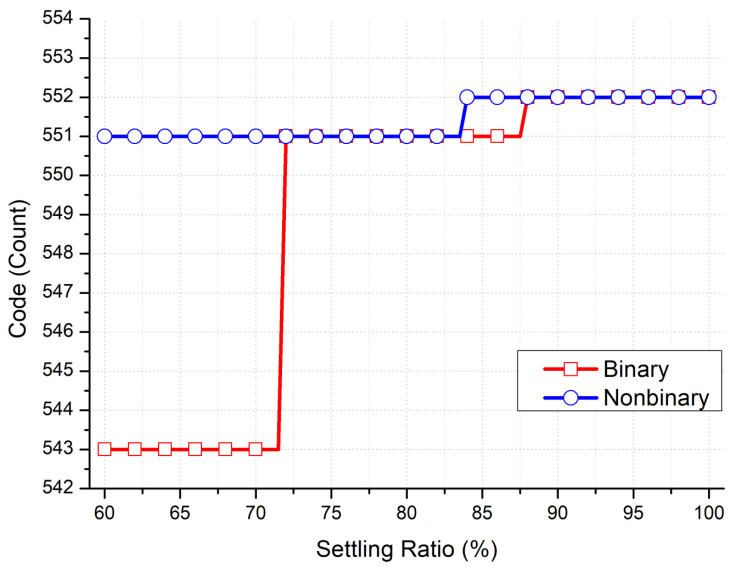
Conversion error versus settling ratio for a radiation detector front-end readout chip containing a binary versus nonbinary SAR ADC with DAC incomplete settling in Matlab simulations.

Figure 3 and Figure 4 present the results of Matlab simulations quantifying the performance of a radiation detection front-end circuit using a nonbinary SAR ADC versus that using a binary SAR ADC. The ideal conversion code count in this simulation is 552, and the result for the binary SAR ADC case is a 1-LSB error, even when the noise effect is only at 0.25 LSB. Conversely, for the nonbinary SAR ADC case, correct conversion results are obtained with up to a 1.5-LSB noise effect. With up to a 7.5-LSB noise effect, the conversion errors for the binary and nonbinary SAR ADCs are 10 and 2 LSB, respectively. With incomplete DAC settling, no conversion errors are found to occur for the binary and nonbinary SAR ADC cases until the settling ratios of 87.5% and 83.8%, respectively, are reached. For the binary SAR ADC case, a 1-LSB conversion error occurs until a settling ratio of 72% is reached, at which point the error increases to an 11-LSB conversion error. By contrast, the nonbinary SAR ADC continues to have a conversion error of only 1 LSB.

Because most applications of radiation detector front-end readout chips require multiple channels, the chip proposed in the present study is a 16-channel front-end readout chip. The use of multiple channels causes a cross-talk effect and reduces the accuracy of an SAR ADC. The cross-talk signal is noise to the SAR ADC, and the SAR ADC thus requires a correction mechanism to reduce the effect of this noise. Section 2 of this paper describes the block diagram of the proposed chip. Section 3 describes how the chip is implemented. Section 4 discusses the chip’s performance in tests. Finally, Section 5 concludes the paper.

## 2. Proposed Structure

**Figure 5:**
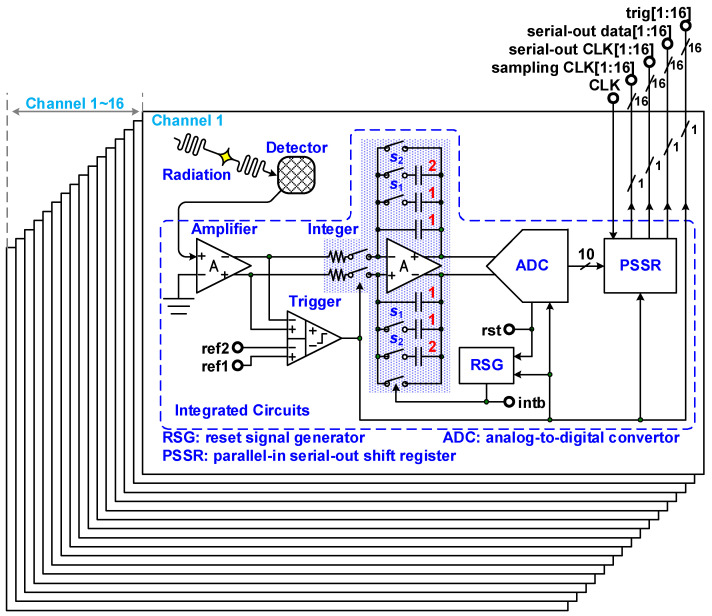
Block diagram of 16-channel front-end readout circuit for a radiation detector.

**Figure 6:**
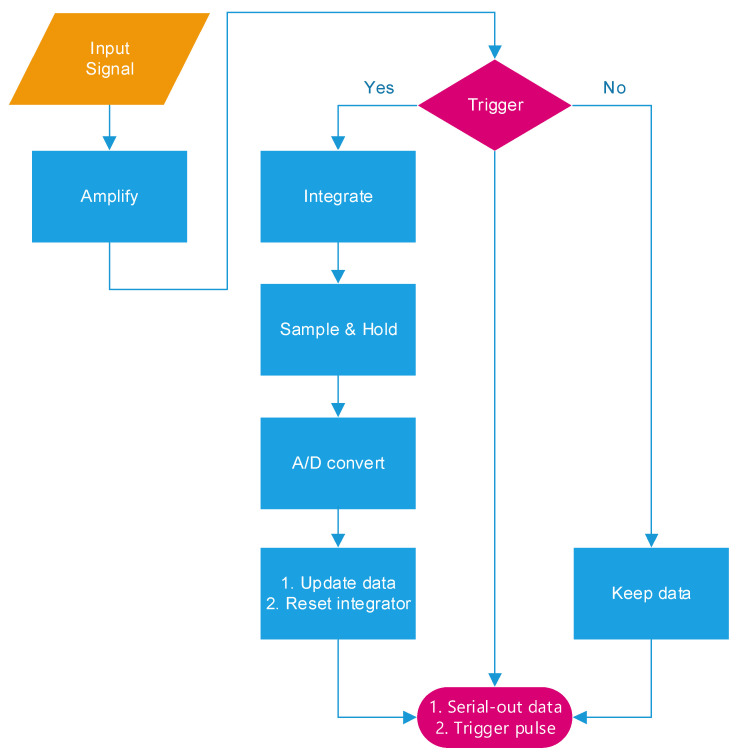
Function flowchart of proposed circuit.

The block diagram of a 16-channel front-end readout circuit for a radiation detector is depicted in Figure 5. The circuit comprises an amplifier, a trigger generator, an integrator, an ADC, a reset signal generator (RSG), a parallel-in serial-out shifter register (PSSR), and a bias circuit. When the radiation detector responds to radiation, a current signal is generated and sent to the amplifier. The amplifier converts the current signal into a voltage signal and transfers this signal to the trigger generator and integrator. The integrator then begins integration, and simultaneously, the ADC begins tracking the integrator’s output signal. The trigger generator is a differential difference comparator and thus has two differential inputs and a single output. The trigger signal is high when the input signal is higher than the reference voltage and low otherwise. When the radiation detector turns off, the trigger generator’s output signal ceases. The ADC then stops its signal tracking and conversion. After it has converted an analog signal, the ADC transfers the parallel digital data to the PSSR and sends a reset signal to the RSG. The RSG resets the integrator after turning off the signal of the trigger generator and the reset signal of the ADC. The amplifier, trigger generator, ADC, and RSG enter into sleep mode until the radiation detector responds again. A flowchart of this process is depicted in Figure 6. The input signal’s CLK periodically controls the PSSR’s renewal of parallel-in digital data from the ADC and transferal of serial-out data, a serial-out clock, and a sampling clock. The sampling clock is the time at which parallel-in digital data should be renewed, whereas the serial-out clock is the time at which serial digital data are read out. Each serial-out datum follows each trigger signal.

The integrator comprises an amplifier, an input resistor, and multiple-integrated-slope switched capacitors. The multiple integrated slopes of these switched capacitors are changed by switches S_1_ and S_2_. The original integrated slope works with one capacitor when the control signals *s*_1_ and *s*_2_ are low. The integrated slope works with two capacitors when the control signals *s*_1_ and *s*_2_ are high and low, respectively. When the control signals *s*_1_ and *s*_2_ are respectively low and high or are both high, the integrated slope works with three or four capacitors. The multiple integrated slopes can cover a substantial input-signal-to-digital-output conversion range and thus support numerous applications.

**Figure 7:**
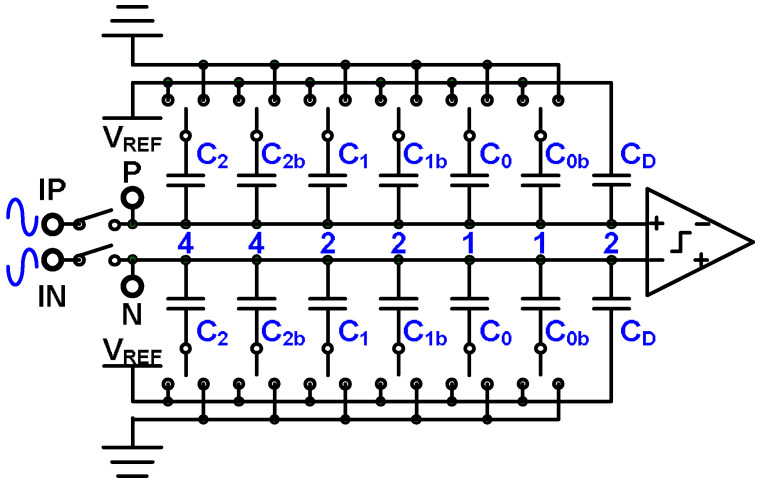
Four-bit binary SAR ADC with split capacitors.

**Figure 8:**
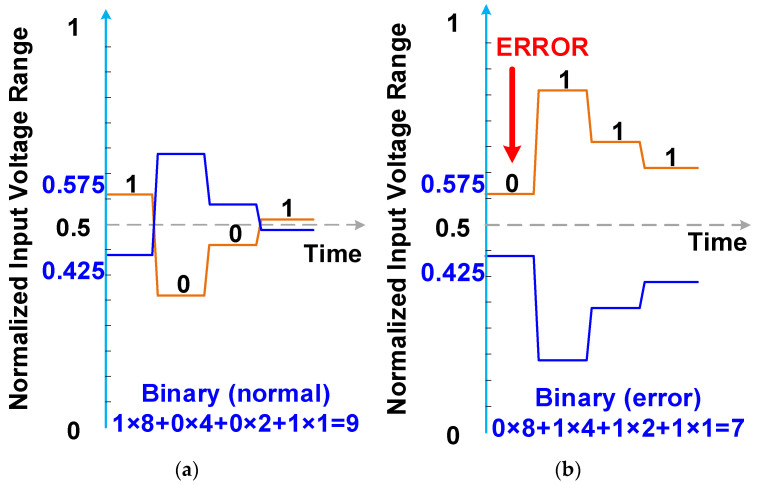
(**a**) Normal conversion process and (**b**) conversion process with MSB error decisions in a 4-bit binary SAR ADC.

A 4-bit binary SAR ADC with a split capacitor DAC [18] is depicted in Figure 7. The power efficiency of the SAR ADC with split capacitor technology is greater than that of a conventional SAR ADC. The capacitors in a conventional 4-bit SAR ADC are denoted C_2_, C_1_, C_0_, and C_D_, and the weighted sizes are 8, 4, 2, and 2. By contrast, in the SAR ADC with split capacitor technology, the DAC structure contains C_2_ and C_2b_ instead of C_2_, C_1_ and C_1b_ instead of C_1_, and C_0_ and C_0b_ instead of C_0_. The normal conversion process of a binary SAR ADC with split capacitors is depicted in Figure 8a, and the conversion process with most significant bit (MSB) error decisions is illustrated in Figure 8b. The digital outputs of the 4-bit binary SAR ADC with split capacitors are *b*_3_, *b*_2_, *b*_1_, and *b*_0_, and the mapping code weights are 8, 4, 2, and 1, respectively. Thus, the digital result is 8 × *b*_3_ + 4 × *b*_2_ + 2 × *b*_1_ + 1 × *b*_0_. The correction results of conversion—*b*_3_, *b*_2_, *b*_1_, and *b*_0_—are 1, 0, 0, and 1, respectively, leading to a calculation result of 9. If an MSB error decision occurs, however, the conversion results are 0, 1, 1, and 1, and the calculation result is 7. The MSB error decision causes an incorrect conversion result.

**Figure 9:**
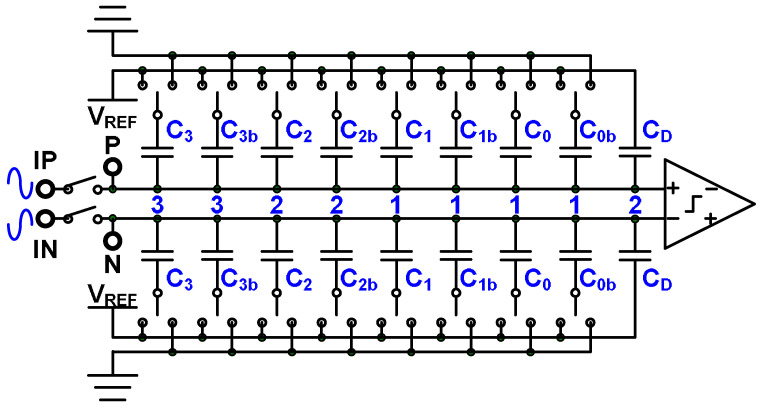
Four-bit nonbinary SAR ADC with split capacitors.

**Figure 10:**
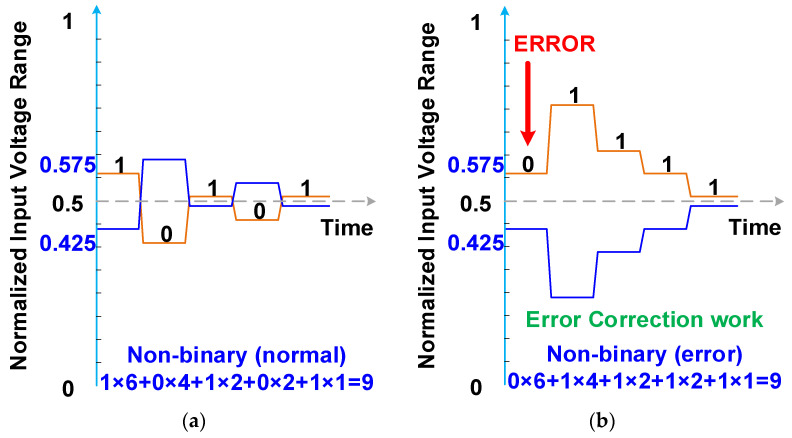
(**a**) Normal conversion process and (**b**) conversion process with MSB error decisions of a 4-bit nonbinary SAR ADC.

A 4-bit nonbinary SAR ADC with split capacitors is depicted in Figure 9. The DAC structure splits two redundant capacitors, C_3_ and C_3b_, to obtain more redundancy code for error correction. The capacitors of the 4-bit nonbinary SAR ADC with split capacitors are C_3_, C_3b_, C_2_, C_2b_, C_1_, C_1b_, C_0_, C_0b_, and C_D_, and their respective weight sizes are 3, 3, 2, 2, 1, 1, 1, 1, and 2. The digital outputs of the 4-bit nonbinary SAR ADC with split capacitors are *b*_4_, *b*_3_, *b*_2_, *b*_1_, and *b*_0_, and the respective mapping code weights are 6, 4, 2, 2, and 1. Thus, the digital result is 6 × *b*_4_ + 4 × *b*_3_ + 2 × *b*_2_ + 2 × *b*_1_ + 1 × *b*_0_. The redundant code weight of *b*_4_ is the summation of the other code weights minus the code weight of *b*_4_ (4 + 2 + 2 + 1 + 1 − 6 = 4). The other redundant code weights are 2, 2, and 0 (calculations: 2 + 2 + 1 + 1 − 2 = 2; 2 + 1 + 1 − 2 = 2; and 1 + 1 − 2 = 0). Because the binary SAR ADC with split capacitors has no redundant capacitors, it has no redundant code weights. The normal conversion process of a 4-bit nonbinary SAR ADC with split capacitors is depicted in Figure 10a. The correction conversion results are 1, 0, 1, 0, and 1, and the calculation result is 9. The conversion process of a 4-bit nonbinary SAR ADC with split capacitors and MSB error decisions is presented in Figure 10b. The conversion results are 0, 1, 1, 1, and 1, and the calculation result is 9. The MSB error decision can be corrected with redundant code weights.

**Figure 11:**
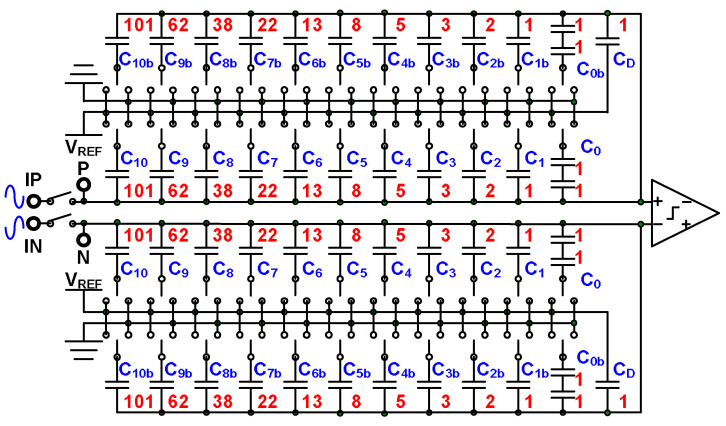
DAC of a nonbinary SAR ADC.

The code weights and redundant code weights of 10-bit binary and nonbinary SAR ADCs are listed in Table 1. When redundant code weights are larger, more redundant bits must be added in the conversion process. In the case of the 10-bit SAR ADC, adding two redundant bits leads to a 20% gain in redundant code weights available for correcting error decisions. The large values of the redundant code weights listed in Table 1 can guarantee larger error correction ranges. Series capacitor technology is employed in this study to reduce the capacitor array of the DAC, which is depicted in Figure 11. The capacitors C_0_ and C_0b_ comprise two series unit capacitors; this means that the size of the capacitor array in the DAC is halved because the equivalent capacitance of this array with two series unit capacitors is a half-unit capacitor [17,19].

## 3. Circuit Design and Implementation

This section describes the circuit structure and how each stage of the block diagram is designed and implemented, from the input signal to the first stage to the output signal from the last stage.

### 3.1. Amplifier

**Figure 12:**
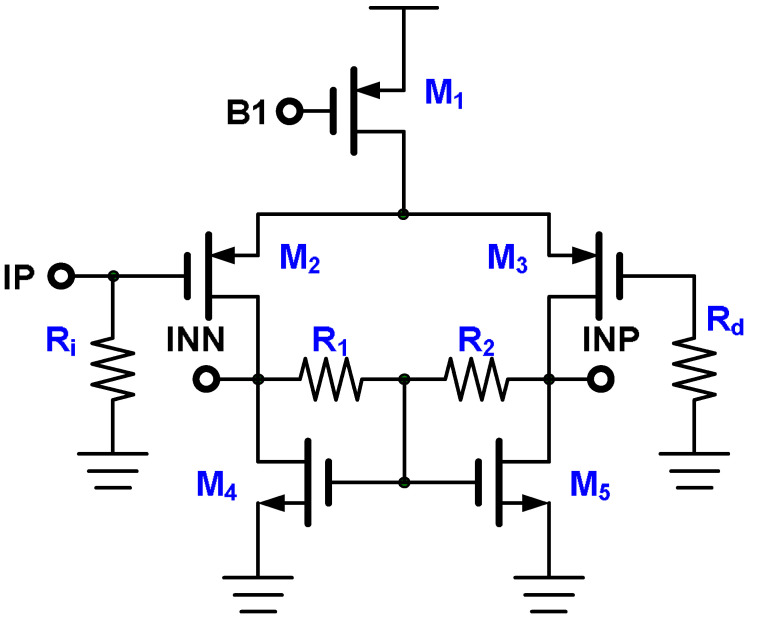
Amplifier.

**Figure 13:**
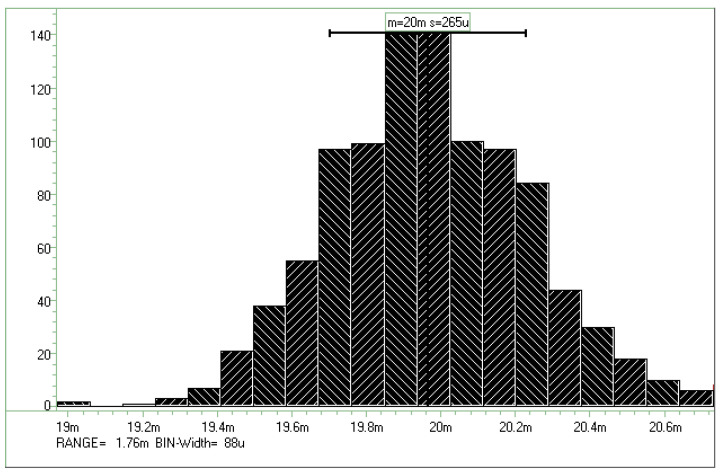
Hspice Monte Carlo simulation of the amplifier with 1000 samples.

The amplifier, which is the signal-receiving stage in the proposed block diagram, has the circuit structure depicted in Figure 12. It comprises input resistors R_i_ and R_d_, a fully differential amplifier M_1_–M_5_, and common feedback resistors R_1_ and R_2_. The current signal is converted into a voltage signal by input resistor R_i_, and the voltage signal is amplified by the amplifier. A trade-off is made between the noise contributed by the input resistance and the range over which the received current signal is converted into the voltage signal. When the input resistance is 1 kΩ, it contributes noise of 13 μV_rms_ when the operating frequency is 10 MHz, as revealed in Equation (1) [20]. Because the converted voltage range for an input current signal of 1 μA to 1 mA is 0.1–1.0 V, the noise contribution of the input resistor can be ignored. In the 16-channel design, the offset between channels must be considered. The large mismatch contribution in the amplifier is the threshold voltage (denoted *V*_TH_) difference between the input pair M_2_ and M_3_. To ensure that the offset tolerance between each channel is acceptable, 1σ < 0.5 mV_rms_, the width and length of the input pair must be sufficiently large, as expressed in Equation (2) [20]. Figure 13 depicts the results of an Hspice Monte Carlo simulation of the amplifier with 1000 samples. The input-referred offset voltage is 0.265 mV_rms_, and the input signal has a voltage of 20 mV.

(1) $$\sqrt{\bar{v^{2}}}=\sqrt{4kTR\Delta f}=\sqrt{1.66\times 10^{-20}\times 1\times 10^{3}\cdot 10\times 10^{6}}\approx 13\;{\mu V}_{\mathrm{rms}}$$

(2) $$\Delta V_{\mathrm{TH}}=\frac{A_{\mathrm{ATH}}}{\sqrt{\mathrm{WL}}}$$

### 3.2. Integrator

**Figure 14:**
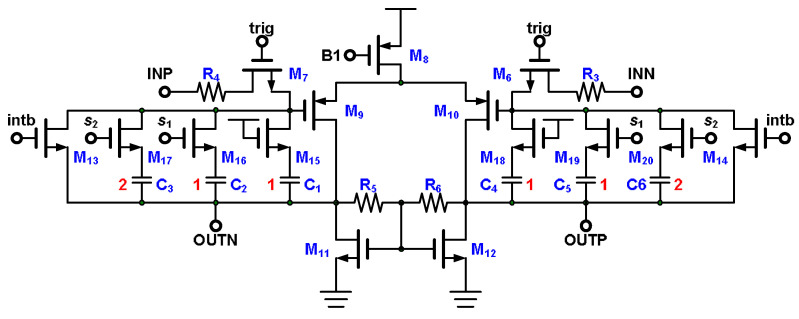
Integrator with multiple switched capacitors.

The integrator (illustrated in Figure 14) comprises the input resistors R_3_ and R_4_, which are switched by M_6_ and M_7_, respectively; a fully differential amplifier M_8_–M_12_; the common feedback resistors R_5_ and R_6_; the reset switches M_13_ and M_14_; and multiple slopes integrated by capacitors C_1_–C_6_ and switches M_15_–M_20_. The integrator integrates when M_6_ and M_7_ turn on and M_13_ and M_14_ turn off, whereas it stops integrating when M_6_, M_7_, M_13_, and M_14_ turn off. Integration is reset when M_6_ and M_7_ turn off and M_13_ and M_14_ turn on. The output voltage of the integrator is integrated by input signal *V*_IN_, switched capacitors C_int_, and input resistors R_4_ and R_6_, as shown in Equation (3). C_int_ comprises C_1_–C_6_. The multiple integrated slopes switch to different integrated slopes when M_15_–M_20_ work in a different mode. When M_15_ and M_18_ always turn on and M_16_, M_17_, M_19_, and M_20_ turn off, the integrated slope is the largest of the multiple integrated slopes. Under this condition, C_int_ is C_1_. For the design, each switched capacitor in the integrator has the same turn-on resistance. In the device, the design size ratio of M_17_:M_16_:M_15_ is 2:1:1. The second integrated slope occurs when M_16_ and M_19_ turn on and M_17_ and M_20_ turn off. C_int_ is then C_1_ + C_2_. The third occurs when M_16_ and M_19_ turn off and M_17_ and M_20_ turn on, in which case C_int_ is C_1_ + C_3_. The last occurs when M_16_–M_17_ and M_19_–M_20_ turn on, resulting in C_int_ being C_1_ + C_2_ + C_3_. Because the capacitance ratio of C_1_ to (C_1_ + C_2_) to (C_1_ + C_3_) to (C_1_ + C_2_ + C_3_) is 1:2:3:4, the ratio between integrated slopes is 12:6:4:3.

(3) $$V_{OUT}{=V}_{OUTP}-V_{OUTN}=-\frac{1}{R_{4}C_{\mathrm{int}}}\int V_{INN}dt+\frac{1}{R_{6}C_{\mathrm{int}}}\int V_{INP}dt=\frac{1}{R_{4}C_{\mathrm{int}}}\int V_{IN}dt$$

### 3.3. Trigger Generator

**Figure 15:**
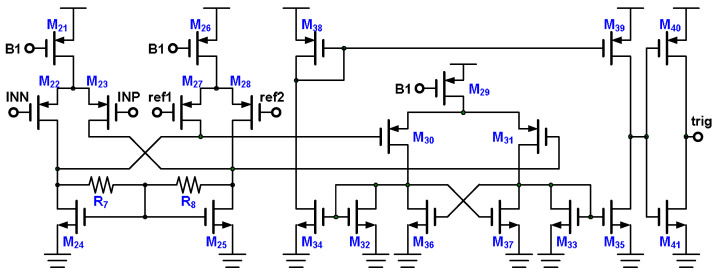
Differential difference input trigger generator.

The trigger generator comprises a differential difference amplifier M_21_–M_28_, R_7_ [21], R_8_, and a comparator M_29_–M_41_, as illustrated in Figure 15. The positive output terminal of the differential difference amplifier is the gate of M_30_, and the negative output terminal is the gate of M_31_. The voltage of the positive output terminal is expressed in Equation (4), the voltage of the negative output terminal is expressed in Equation (5), and the differential voltage of the output terminal is expressed in Equation (6). Using the result of the differential difference amplifier, the comparator generates a trigger signal.

(4) $$v_{op}=-\left(v_{INN}\times g_{mp}+v_{ref1}\times g_{mp}\right)\times r_{on}$$

(5) $$v_{on}=-\left(v_{INP}\times g_{mp}+v_{ref2}\times g_{mp}\right)\times r_{on}$$

(6) $$v_{op}-v_{on}=\left[\left(v_{INP}-v_{INN}\right)-\left(v_{ref1}-v_{ref2}\right)\right]\times g_{mp}\times r_{on}$$

The comparator comprises differential inputs (M_30_ and M_31_), a cross-coupled latch (M_36_ and M_37_), a diode-connected load (M_33_ and M_34_), a current summary (M_34_, M_35_, M_38_, and M_39_), and an inverter-based output buffer (M_40_ and M_41_). When the gate voltage of M_30_ is higher than that of M_31_, the drain current of M_30_ is lower than that of M_31_; the cross-coupled latch then reduces the drain voltage of M_30_ and increases the drain voltage of M_31_. The current summary part summarizes the drain currents of the diode-connected loads M_32_ and M_33_, and the inverter-based output buffer increases the level of the output voltage to the supply voltage level. If the gate voltage of M_30_ is lower than that of M_31_, the level of the output voltage of the comparator decreases to the ground voltage level.

### 3.4. Bias Circuit

**Figure 16:**
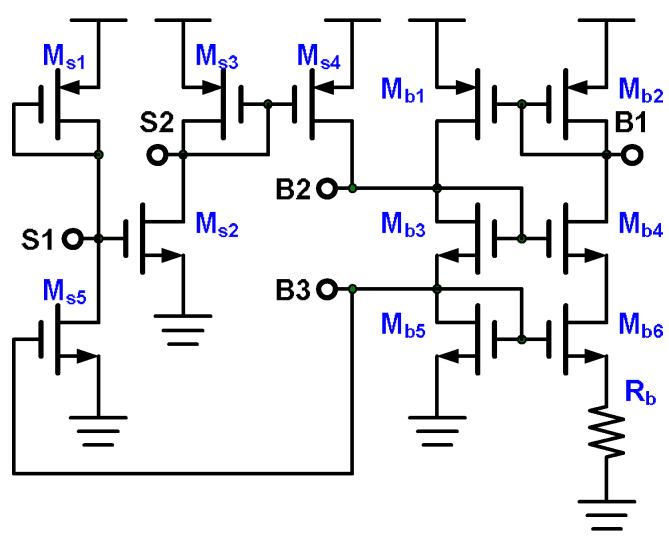
Bias circuit.

The bias circuit, depicted in Figure 16 [20], has a bias part and a start-up part. Because the bias part comprises P-type and N-type current mirror structures of M_b1_–M_b6_ and R_b_, the circuit operates in two static states. One static state involves current, whereas the other does not. The circuit requires a start-up circuit to push the bias part into the correct operation mode. When the supply voltage turns on and the bias part is in the incorrect operation mode, the voltages *V*_B2_ and *V*_B3_ are low. When *V*_B3_ is low, M_s5_ turns off, the diode-connected M_s1_ turns on M_s2_, and M_s2_ and the diode-connected M_s3_ turn on M_s4_ to increase *V*_B2_ and *V*_B3_ to the correct operation voltage. Because *V*_B3_ is in the correct operation mode, M_s5_ turns on and M_s2_ turns off, and M_s4_ turns off. The start-up part turns off, and the bias part is then operating in the correct mode. The bias circuit biases the amplifier depicted in Figure 12, the integrator displayed in Figure 14, and the trigger generator illustrated in Figure 15. To ensure favorable matching of the bias device M_b2_, the amplifier’s M_1_, the integrator’s M_8_, and the trigger generator’s M_21_, M_26_, and M_29_ must have the same width, length, symmetry, and device layout direction, but the device multiple can be different. Without using resistor R_b_, the current mirror loop involving M_b1_–M_b6_ gives positive feedback. Adding the resistor R_b_ and the multiple M_b6_ results in the loop giving negative feedback. The bias current *I*_b2_ is determined by the resistance R_b_ and the size of M_b5_–M_b6_, as expressed in Equation (7) [20]. The bias current *I*_b2_ has extremely low sensitivity to supply voltage variation and noise.

(7) $$I_{b2}=\frac{2}{\mu_{n}C_{ox}{\left(W/L\right)}_{b5}}\cdot \frac{1}{R_{b}}\left(1-\frac{1}{\sqrt{{{\left(W/L\right)}_{b6}/\left(W/L\right)}_{b5}}}\right)$$

### 3.5. Reset Circuit

**Figure 17:**
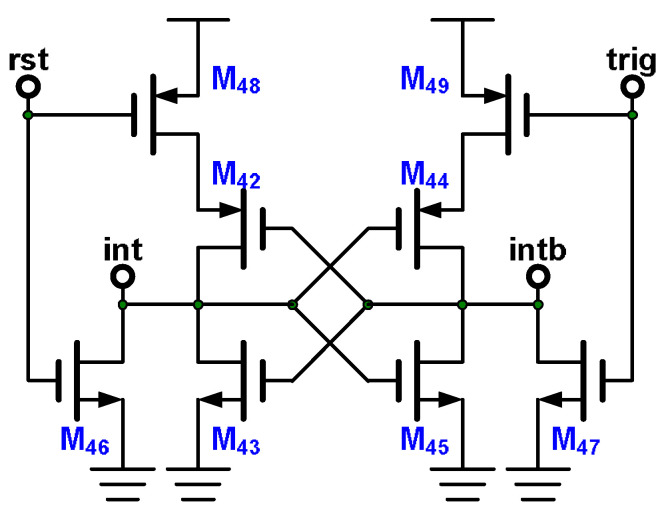
Reset circuit.

The integrator illustrated in Figure 14 starts to integrate when it receives the trigger signal and retains the result until the SAR ADC has completed its conversion. The reset circuit displayed in Figure 17 combines the trigger signal from the trigger circuit with the reset signal from the SAR ADC and then generates the reset signals int and intb, which reset the integrator. The reset circuit operates in four states: reset, hold after reset, trigger, and hold after trigger. In the reset state, a reset signal pulse enters node rst. And the node trig is 0. M_43_, M_44_, M_46_, and M_49_ turn on, and the others turn off. The node int changes to 0, and the node intb changes to 1. In the hold after reset state, the nodes rst and trig are 0, and M_46_–M_47_ turn off. M_42_–M_45_ and M_48_–M_49_ comprise a latch circuit and keep the int and intb voltage levels at 0 and 1, respectively. In the trigger state, a trigger signal pulse is sent to the node trig. In the meantime, the node rst is 0. M_42_, M_45_, M_47_, and M_48_ turn on, and the others turn off. The node int changes to 1, and the node intb changes to 0. In the hold after trigger state, the latch circuit keeps the int and intb voltage levels at 1 and 0, respectively.

### 3.6. Nonbinary SAR ADC

**Figure 18:**
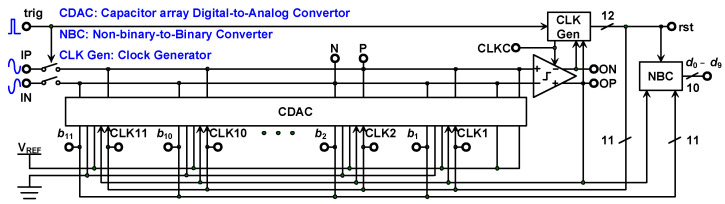
Nonbinary SAR ADC block diagram.

The block diagram of the SAR ADC is presented in Figure 18. The SAR ADC comprises a comparator, a clock generator, a capacitor array DAC (CDAC), and a nonbinary-to-binary converter (NBC). The clock generator is initiated when the falling edge of the trigger signal is reached and generates the timing clock that controls the CDAC, comparator, and NBC. The comparator detects the difference in voltage between the positive terminal and negative terminal of the CDAC. The CDAC follows the signal from the clock generator and comparator to switch the capacitors; the weight size of the capacitors is depicted in Figure 11. The data output from the ADC to the PSSR input are 10b binary code, which is depicted in Equation (8).

(8) $$\begin{array}{ll} ADC_{OUT}= & 512\cdot d_{9}+256\cdot d_{8}+128\cdot d_{7}+64\cdot d_{6}+32\cdot d_{5}\\ & +16\cdot d_{4}+8\cdot d_{3}+4\cdot d_{2}+2\cdot d_{1}+1\cdot d_{0} \end{array}$$

### 3.7. NBC

**Figure 19:**
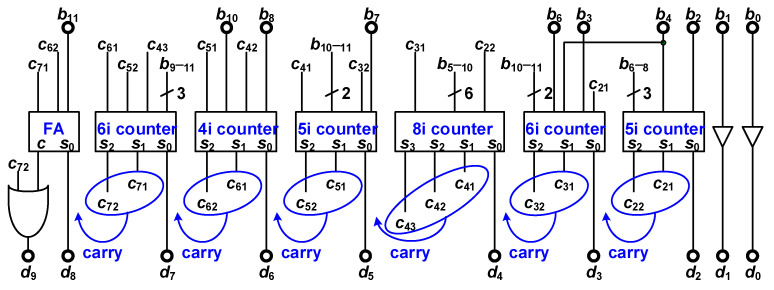
NBC of nonbinary SAR ADC.

The NBC, illustrated in Figure 19, comprises many counters that convert the nonbinary comparator results into binary digital code. The input of the NBC is depicted in Equation (9). Each piece of nonbinary code can be split into binary parts; for example, 404 × *b*_11_, 248 × *b*_10_, 152 × *b*_9_, 88 × *b*_8_, 52 × *b*_7_, 20 × *b*_5_, and 12 × *b*_4_ can be split into (256 + 128 + 16 + 4) × *b*_11_, (128 + 64 + 32 + 16 + 8) × *b*_10_, (128 + 16 + 8) × *b*_9_, (64 + 16 + 8) × *b*_8_, (32 + 16 + 4) × *b*_7_, (16 + 4) × *b*_5_, and (8 + 4) × *b*_4_, respectively. The nonbinary code is split into binary parts. Counters are then employed to summarize the same-order binary parts. The results of the counter are a binary result and carry-out data. The carry-out data must be summarized with the next-order binary code. Because the comparator results for *b*_0_ and *b*_1_ are binary, they do not need to be converted. In the last stage, an OR gate is used to obtain a summary of the carry-out data (denoted *c*_72_) and the carry-out data of the full adder [17].

(9) $$\begin{array}{ll} NBC_{IN}= & 404\cdot b_{11}+248\cdot b_{10}+152\cdot b_{9}+88\cdot b_{8}+52\cdot b_{7}+32\cdot b_{6}\\ & +20\cdot b_{5}+12\cdot b_{4}+8\cdot b_{3}+4\cdot b_{2}+2\cdot b_{1}+1\cdot b_{0} \end{array}$$

### 3.8. PSSR

**Figure 20:**
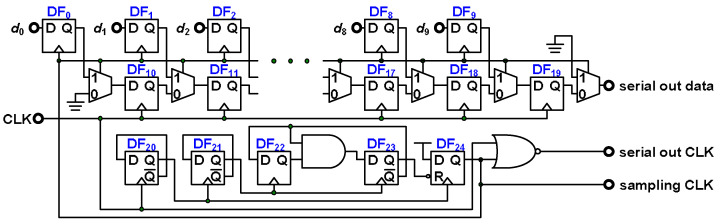
PSSR.

Because the application has 16 channels, the area cost of the parallel-out data solution is enormous, and the data transmission line is overly large. This study employs serial-out data to prevent those problems. The PSSR illustrated in Figure 20 is used to convert the data from the parallel-in to the serial-out form. An off-chip clock is required to control the PSSR. An AND gate and the D-flip-flops (DFFs) DF_20_–DF_23_ divide the input clock by 12, and DF_24_ generates the sampling clock for loading parallel data. The NOR gate generates the serial-out clock for off-chip digital I/O reception of serial-out data. The PSSR stores data in parallel by using DFFs DF_0_–DF_9_ and transfers the serial-out data by using multiplexes and DFFs DF_10_–DF_19_.

## 4. Measurement Results

**Figure 21:**
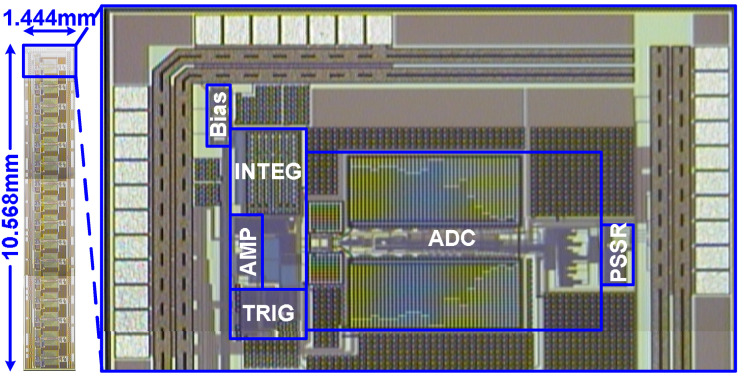
Die photograph.

**Figure 22:**
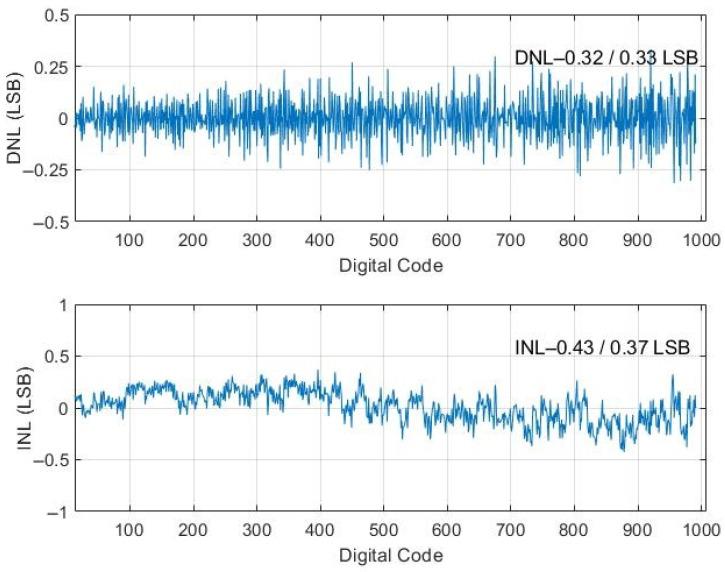
Static-state performance of nonbinary SAR ADC.

**Figure 23:**
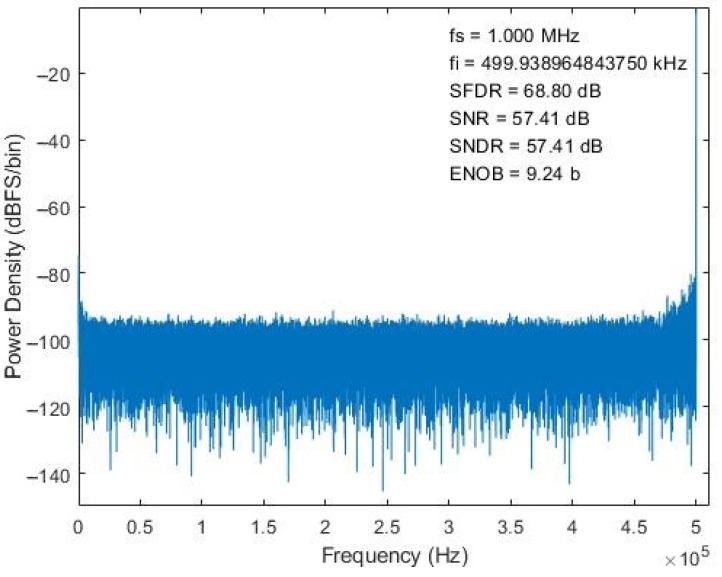
Dynamic performance of nonbinary SAR ADC.

The designed chip is implemented with CMOS 0.18 μm 1P6M technology. A photograph of the die is presented in Figure 21. The chip is 1.444 mm × 10.568 mm and contains a 16-channel front-end readout circuit for a radiation detector. Regarding the chip’s static-state performance, the minimum differential nonlinearity (DNL) and integral nonlinearity (INL) of the nonbinary SAR ADC are −0.32 and −0.43 LSB, whereas the maxima of these indicators are 0.33 and 0.37 LSB, respectively, as revealed in Figure 22. Regarding the chip’s dynamic performance, the spurious-free dynamic range, signal-to-noise ratio, signal-to-noise-and-distortion ratio, and effective number of bits (ENOB) of the nonbinary SAR ADC when a 500-KHz sine signal with a 1 MS/s sampling rate is input are 68.8 dB, 57.41 dB, 57.41 dB, and 9.24 b, respectively, as detailed in Figure 23. The power consumption of the SAR ADC is 23.5 μW. The figure of merit (FoM) is calculated using Equation (10) from [22]. The FoM of the SRA ADC is 38.9 fJ/conversion step.

(10) $$FoM_{W}=\frac{Power}{2^{ENOB}\times f_{Sampling}}\;J/conv.-step$$

**Figure 24:**
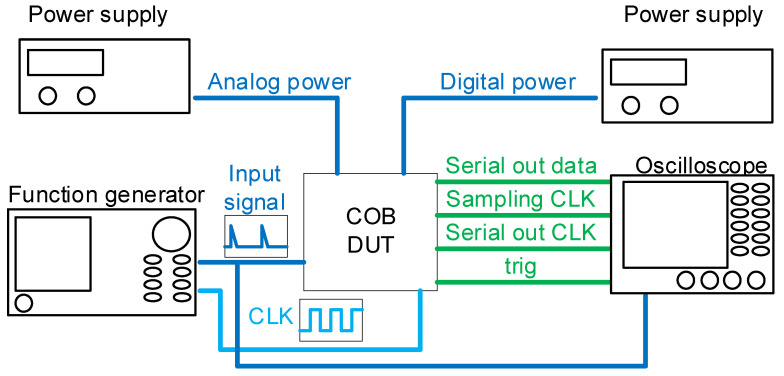
Measurement environment.

The environment used for measurement in this study is depicted in Figure 24. The input signal of the chip on board device under test is provided by a function generator. The output signal is input to an oscilloscope. Analog power and digital power are supplied by respective power supplies.

**Figure 25:**
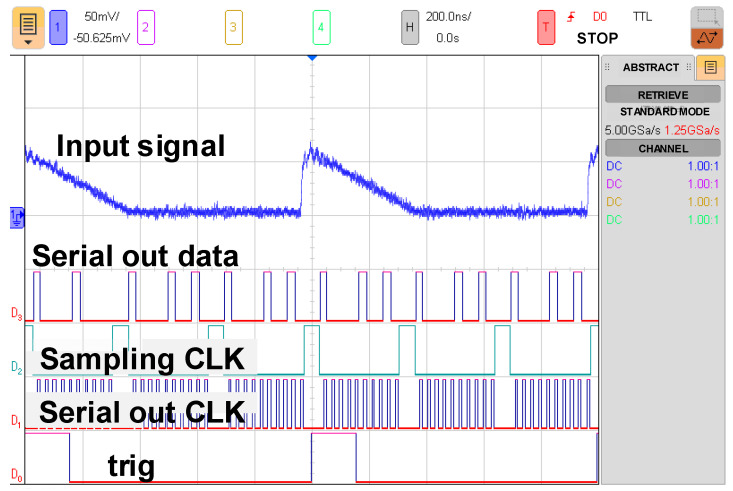
Output signal on an oscilloscope.

**Figure 26:**
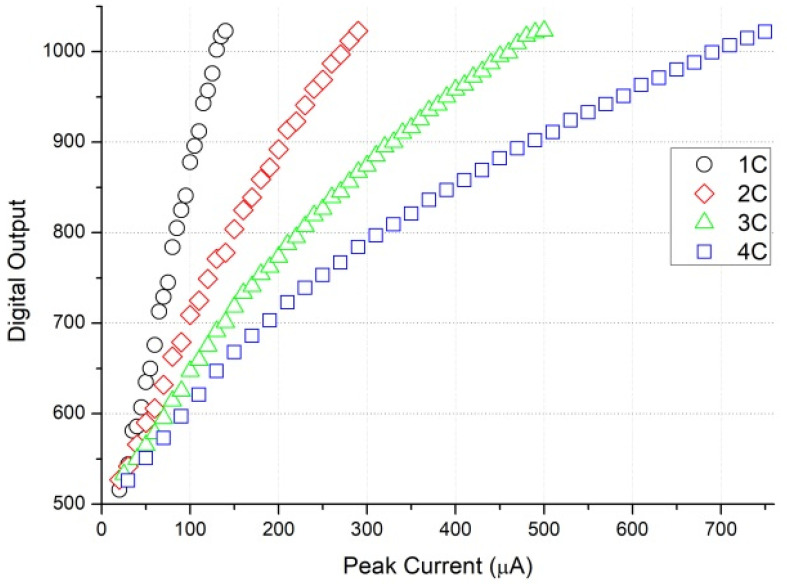
Peak current to digital data with different integration coefficients.

**Figure 27:**
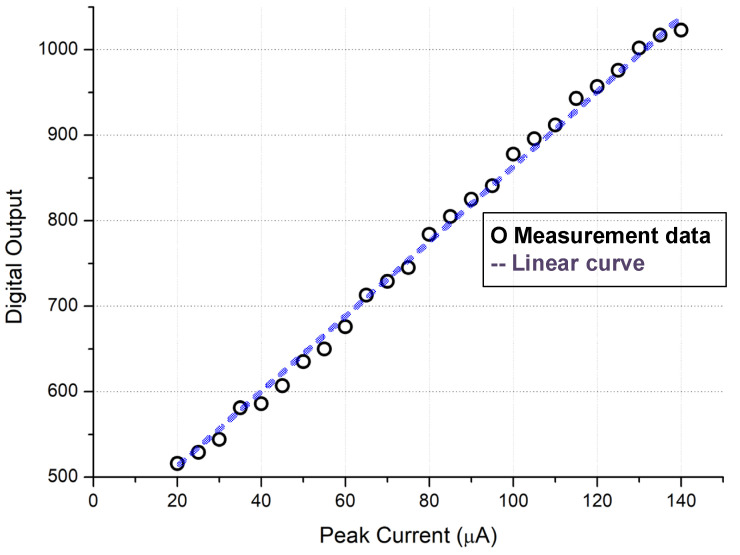
Conversion curve from input peak current to the digital output.

**Figure 28:**
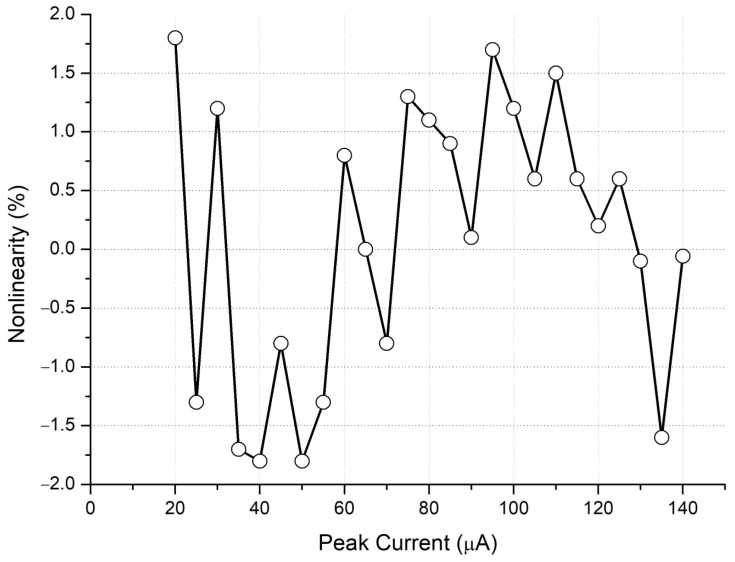
Nonlinearity of conversion curve from input peak current to digital output.

**Figure 29:**
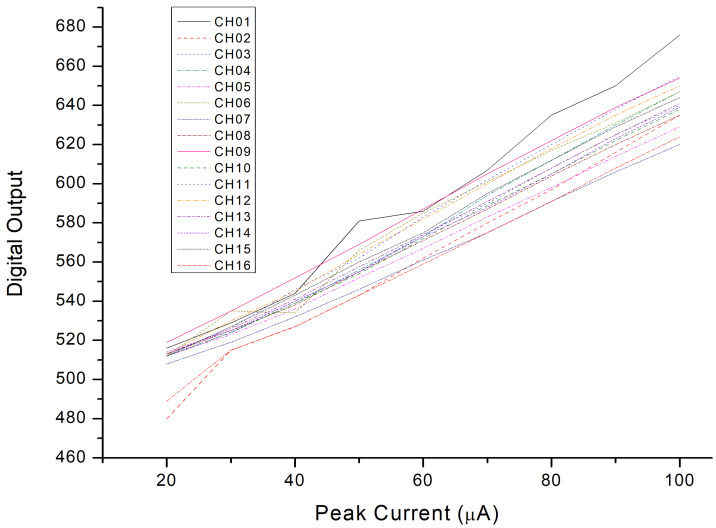
Conversion curves of 16 channels from input peak current to digital output.

**Figure 30:**
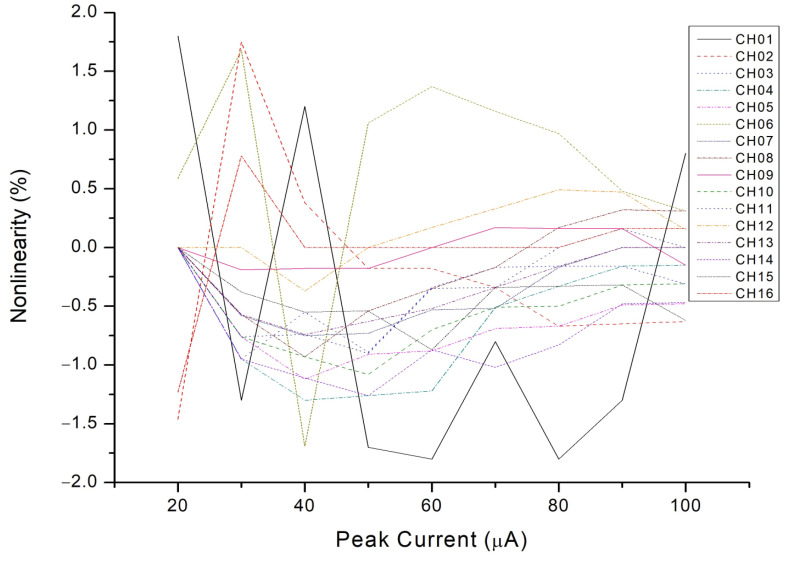
Nonlinearity of conversion curves of 16 channels from input peak current to digital output.

The entire chip test is depicted in Figure 25. When the input signal rises and falls, the trigger signal turns on and off, respectively. The serial-out data transfer the detection result with the sampling clock and the serial-out clock. Conversion curves with different integrated slopes are displayed in Figure 26. The maximum cover range of the peak-to-peak current pulse is 750 μA. The resolution of the conversion curve and the minimum coverage of testing, described in Figure 27, are 208.3 nA and 20 μA, respectively. When the shaping time is 200 ns, the input dynamic is 2–75 pC. The nonlinearity of the conversion curve versus the peak current in the digital output is depicted in Figure 28; the maximum nonlinearity is 1.8%. The conversion curves of 16 channels from the input peak current to the digital output are depicted in Figure 29. Because of the process variation of resistors and capacitors in the integrator, the slope for each channel is different. The nonlinearity of the aforementioned conversion curves is depicted in Figure 30. The maximum nonlinearity is found to be 1.8%.

**Figure 31:**
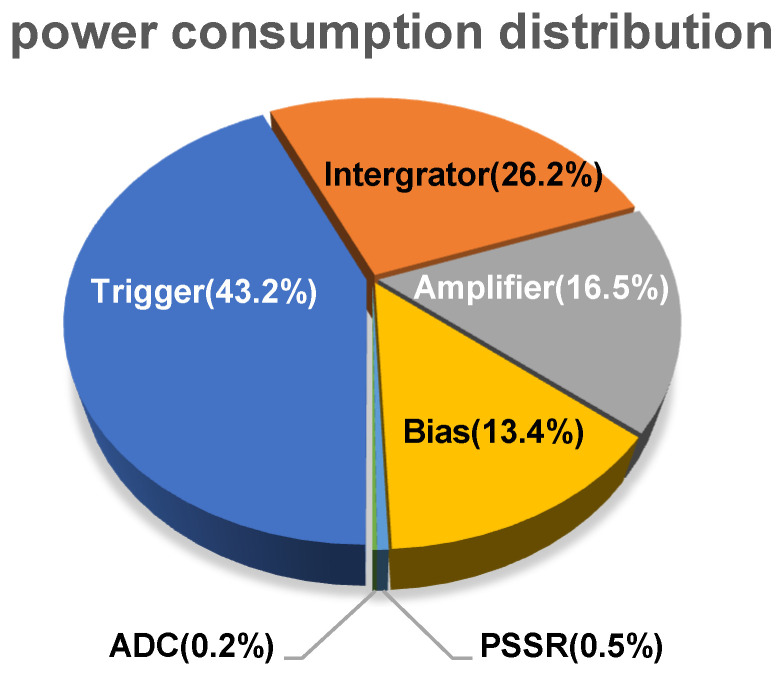
Power consumption distribution.

The power consumption is 19 mW/channel for a 3-V supply voltage. The power consumption of the amplifier, integrator, trigger, ADC, and PSSR is 3.14, 4.98, 8.20, 0.03, and 0.1 mW, respectively. The proportion of power consumed by each block is depicted in Figure 31.

The performance of the proposed design and that of other designs [11,12,13,23,24] are summarized in Table 2. The number of channels in the proposed design is 16, which is higher than the number in the previously reported designs [11,13,23,24]. The input charge range, 2/75 pC, is larger than that in three other designs [13,23,24]. The nonlinearity of the conversion curve, 1.8%, is smaller than that in two other designs [11,23] and close to that in another [13]. The sampling rate per channel, 1 MS/s, is better than that in two other designs [12,23] and the same as that in another [13]. The DNL and INL in the present study—in the ranges −0.32 to 0.33 and −0.43 to 0.37, respectively—are better than those in two other designs [11,12,13]. The resolution of the ADC, 10 b, is better than that in two other designs [12,13]. The ENOB, 9.24 b, is better than that in one other design [12]. The ADC power consumption per channel, 23.5 μW, is better than that in one other design [12] and close to that in another [13]. The FoM of the proposed ADC, 0.0389 pJ/conversion step, is better than that in another design [12].

## 5. Conclusions

To scale down a radiation detector such that it can be incorporated into a wearable device, in this study, integrated circuit technology was employed to fabricate a 16-channel front-end readout chip for such a detector, and chip performance tests were conducted. The DNL, INL, ENOB, and power consumption of the proposed ADC are −0.32 to 0.33, −0.43 to 0.37, 9.24 b, and 23.5 μW, respectively. The resolution is 208.3 nA, and the cover range of the input current pulse from peak to peak is 20–750 μA with multiple integrated slopes. The equal input dynamic range is 2–75 pC, and the maximum nonlinearity is 1.8%. This chip is suitable for use in radiation detection, the IoT, and wearable biomedical applications.

## Figures and Tables

**Table 1:** Bit weights of binary and nonbinary SAR ADCs.

Bit	Binary Code Weight	Redundant Code Weight	Nonbinary Code Weight	Redundant Code Weight
*b*_11_	-	-	404	216
*b*_10_	-	-	248	124
*b*_9_	512	0	152	68
*b*_8_	256	0	88	44
*b*_7_	128	0	52	28
*b*_6_	64	0	32	16
*b*_5_	32	0	20	8
*b*_4_	16	0	12	4
*b*_3_	8	0	8	0
*b*_2_	4	0	4	0
*b*_1_	2	0	2	0
*b*_0_	1	0	1	0
Dummy	1	0	1	0

**Table 2:** Summary of performance of proposed and other designs.

	This Work	[11]	[12]	[13]	[23]	[24]
Technology	0.18 μM	0.35 μM	0.18 μM	0.18 μM	-	0.35 μM
Supply voltage (V)	3/1 V	3.3	3.3	1.8	±15/3 V	5/1 V
Power per channel (mW)	19	15	15	0.02	-	30
Number of channels (unit)	16	10	64	1	1	8
Shaping time (ns)	200	280	300	100	10,000	5/10
Input charge range (pC)	2/75	2.4/104	0.48/520	0.25/17	0.01/33	0/3
Nonlinearity of converting curve (%)	1.8	<3		1.7	5	-
ADC type	SAR	-	Pipeline	Integrated	SAR	TDC
Sampling rate per channel (MS/s)	1	-	0.39	1	20 k	
DNL of ADC (LSB)	−0.32/0.33	-	−0.62/0.67	−0.36/0.12	-	
INL of ADC (LSB)	−0.43/0.37	-	−0.39/0.72	−0.38/0.5	-	
Resolution of ADC (b)	10	-	8	8	12	40 psec
ENOB of ADC (b)	9.24	-	6.03	-	-	
ADC power per channel (μW)	23.5	-	390.63	20	-	-
FoM of ADC (pJ/conversion step)	0.0389	-	15.303	-	-	-

## Data Availability

Data are contained within the article.

## References

[1] Hsu W.-Y., Cheng Y.-W. (2023). EEG-Channel-Temporal-Spectral-Attention Correlation for Motor Imagery EEG Classification. IEEE Trans. Neural Syst. Rehabil. Eng..

[2] Ozkan H., Ozhan O., Karadana Y., Gulcu M., Macit S., Husain F. (2020). A Portable Wearable Tele-ECG Monitoring System. IEEE Trans. Instrum. Meas..

[3] Teferra M.N., Hobbs D.A., Clark R.A., Reynolds K.J. (2022). Electronic-Textile 12-Lead Equivalent Diagnostic Electrocardiogram Based on the EASI Lead Placement. IEEE Sens. J..

[4] Quiroz-Juárez M.A., Jiménez-Ramírez O., Vázquez-Medina R., Ryzhii E., Ryzhii M., Aragón J.L. (2018). Cardiac Conduction Model for Generating 12 Lead ECG Signals With Realistic Heart Rate Dynamics. IEEE Trans. Nanobiosci..

[5] Yang W., Wang S. (2022). A Privacy-Preserving ECG-Based Authentication System for Securing Wireless Body Sensor Networks. IEEE Internet Things J..

[6] Bortolotti D., Mangia M., Bartolini A., Rovatti R., Setti G., Benini L. (2018). Energy-Aware Bio-Signal Compressed Sensing Reconstruction on the WBSN-Gateway. IEEE Trans. Emerg. Top. Comput..

[7] Morahan A.J., D’Adda I., Erlandsson K., Carminati M., Rega M., Walls D., Fiorini C., Hutton B.F. (2023). Challenges in Acquiring Clinical Simultaneous SPECT-MRI on a PET-MRI Scanner. IEEE Trans. Radiat. Plasma Med. Sci..

[8] Zhang Z., Yu Q., Zhang Q., Li J., Wu K., Ning N. (2022). A Code-Recombination Algorithm-Based ADC With Feature Extraction for WBSN Applications. IEEE Trans. Very Large Scale Integr. (VLSI) Syst..

[9] Surti S., Werner M.E., Perkins A.E., Kolthammer J., Karp J.S. (2007). Performance of Philips Gemini TF PET/CT scanner with special consideration for its time-of-flight imaging capabilities. J. Nucl. Med..

[10] Muzic R.F., Kolthammer J.A. (2006). PET performance of the Gemini TF: A time-of-flight PET/CT scanner. Proc. IEEE Nucl. Sci. Symp. Conf. Rec..

[11] Ollivier-Henry N., Gao W., Mbow N.A., Brasse D., Humbert B., HuGuo C., Colledani C., Hu Y. (2011). Design and Characteristics of a Full-Custom Multichannel Front-End Readout ASIC Using Current-Mode CSA for Small Animal PET Imaging. IEEE Trans. Biomed. Circuits Syst..

[12] Gao W., Gao D., Wei T., Zeng H., Duan Y., Lu S., Shen L., Xu W., Xie Q., Hu Y. Design of a Monolithic Multi-Channel Front-End Readout ASIC for LYSO/SiPM-based SmallAnimal Flat-Panel PET Imaging. Proceedings of the 2011 IEEE Nuclear Science Symposium Conference Record.

[13] Schemm N., Balkır S., Hoffman M.W. (2010). A 4-μW CMOS Front End for Particle Detection Applications. IEEE Trans. Circuits Syst.—II Express Briefs.

[14] Gao W., Gao D., Hu-Guo C., Wei T., Hu Y. (2011). Design and Characteristics of an Integrated Multichannel Ramp ADC Using Digital DLL Techniques for Small Animal PET Imaging. IEEE Trans. Nucl. Sci..

[15] Braga L.H.C., Gasparini L., Grant L., Henderson R.K., Massari N., Perenzoni M., Stoppa D., Walker R. (2014). A Fully Digital 8 × 16 SiPM Array for PET Applications with Per-Pixel TDCs and Real-Time Energy Output. IEEE J. Solid-State Circuits.

[16] Kuttner F. A 1.2V 10b 20MSample/s Non-Binary Successive Approximation ADC in 0.13μm CMOS. Proceedings of the 2002 IEEE International Solid-State Circuits Conference, Digest of Technical Papers (Cat. No.02CH37315).

[17] Qiu L., Wang K., Yang C., Zheng Y. (2020). A Low Power Pre-Setting Based Sub-Radix-2 Approximation for Multi-bit/cycle SAR ADCs. IEEE Access.

[18] Ginsburg B.P., Chandrakasan A.P. (2007). 500-MS/s 5-bit ADC in 65-nm CMOS with Split Capacitor Array DAC. IEEE J. Solid-State Circuits.

[19] Zhang Q., Ning N., Li J., Yu Q., Zhang Z., Wu K. (2020). A High Area-Efficiency 14-bit SAR ADC with Hybrid Capacitor DAC for Array Sensors. IEEE Trans. Circuits Syst. I Regul. Pap..

[20] Razavi B. (2006). Design of Analog CMOS Integrated Circuit.

[21] Mahmoud S.A., Soliman A.M. (1998). Soliman. The Differential Difference Operational Floating Amplifier: A New Block for Analog Signal Processing in MOS Technology. IEEE Trans. Circuits Syst. II Analog Digit. Signal Process..

[22] Verma N., Chandrakasan A.P. (2007). An Ultra Low Energy 12-bit Rate-Resolution Scalable SAR ADC for Wireless Sensor Nodes. IEEE J. Solid-State Circuits.

[23] Pettinato S., Orsini A., Girolami M., Trucchi D.M., Rossi M.C., Salvatori S. (2019). A High-Precision Gated Integrator for Repetitive Pulsed Signals Acquisition. Electronics.

[24] Gómez S., Sánchez D., Mauricio J., Picatoste E., Sanuy A., Sanmukh A., Ribó M., Gascón D. (2021). Multiple Use SiPM Integrated Circuit (MUSIC) for Large Area and High Performance Sensors. Electronics.

